# Association of Daily Doses of Buprenorphine With Urgent Health Care Utilization

**DOI:** 10.1001/jamanetworkopen.2024.35478

**Published:** 2024-09-25

**Authors:** Sarah Axeen, Rosalie Liccardo Pacula, Jessica S. Merlin, Adam J. Gordon, Bradley D. Stein

**Affiliations:** 1 Schaeffer Center for Health Policy and Economics, University of Southern California, Los Angeles; 2 Department of Emergency Medicine, Keck School of Medicine of USC, Los Angeles, California; 3 Department of Medicine, University of Pittsburgh School of Medicine, Pittsburgh, Pennsylvania; 4 Program for Addiction Research, Clinical Care, Knowledge and Advocacy, Division of Epidemiology, Department of Internal Medicine, University of Utah School of Medicine, Salt Lake City; 5 Informatics, Decision-Enhancement, and Analytic Sciences Center, VA Salt Lake City Health Care System, Salt Lake City, Utah; 6 Department of Health Policy and Management, Sol Price School of Policy, University of Southern California, Los Angeles; 7 RAND, Pittsburgh, Pennsylvania

## Abstract

**Importance:**

Higher buprenorphine doses may benefit the increasing number of individuals using fentanyl and other synthetic opioids, but there is little empirical evidence on the efficacy of such higher doses.

**Objective:**

To examine the association between higher buprenorphine doses (above 16 mg and 24 mg) and subsequent emergency department (ED) or inpatient service use among patients diagnosed with opioid use disorder.

**Design, Setting, and Participants:**

This cross-sectional study was a retrospective analysis of health data from Optum’s deidentified Clinformatics Data Mart Database from 2016 to 2021 for commercially insured individuals aged 18 years or older diagnosed with opioid use disorder (OUD). Eligible participants initiated buprenorphine after at least 90 days of enrollment and were dispensed at least a 14-day supply of buprenorphine. Data were analyzed from September 2023 through February 2024.

**Exposures:**

Maximum buprenorphine dose received by a patient for 14 or more days: more than 24 mg, more than 16 mg to 24 mg, more than 8 mg to 16 mg, or 1 mg to 8 mg.

**Main Outcomes and Measures:**

Days from initiation of the maximum buprenorphine dose to an ED or inpatient visit for a behavioral health diagnosis, controlling for patient demographics, comorbid conditions, time to reaching maximum dose, buprenorphine discontinuation, and pre-buprenorphine health care utilization.

**Results:**

A total of 35 451 individuals with an OUD diagnosis who began buprenorphine treatment were identified (mean [SD] age, 46.2 [15.1] years; 20 983 male [59.2%]; 3326 Black [9.4%], 2411 Hispanic [6.8%], 26 712 White [75.3%]). The most common dose was more than 8 mg to 16 mg daily (14 802 patients [42.9%]), with 9669 patients (27.3%) in the 1 mg to 8 mg tier, 10 329 patients (29.1%) in the 8 mg to 16 mg tier, and 651 patients (1.8%) in the tier receiving more than 24 mg. Among all patients receiving buprenorphine, 12.5% experienced an ED or inpatient visit. Survival analysis shows patients receiving doses more than 24 mg and between 16 mg to 24 mg had longer times to ED or inpatient use than patients receiving from 8 mg to 16 mg (time ratio [TR], 1.11; 95% CI, 1.02 to 1.20) and more than 24 mg (TR, 1.37; 95% CI, 1.04 to 1.81). Findings for doses above 16 mg daily were consistent for observation windows as short as 365 days (more than 24 mg: TR, 1.48; 95% CI, 1.01-2.18; more than 16 mg to 24 mg: TR, 1.19; 95% CI, 1.06-1.32).

**Conclusions and Relevance:**

These findings contribute to the sparse empirical research regarding potential benefits of higher-dose buprenorphine treatment of individuals with OUD. Clinicians should be aware of the potential effects of higher buprenorphine doses on health care utilization while policymakers work to ensure equitable access to individuals who could potentially benefit from higher doses.

## Key Points

**Question:**

Is buprenorphine treatment using doses higher than Food and Drug Administration recommendations associated with subsequent acute health care utilization?

**Findings:**

In this cross-sectional study using health care claims data from 35 451 US adults with an opioid use disorder diagnosis, those receiving higher maximum doses of buprenorphine (ie, doses above 16 mg and 24 mg) had significantly lower rates of acute care utilization than their peers receiving FDA-recommended doses (between 8 mg and 16 mg).

**Meaning:**

These results suggest that higher doses of buprenorphine are associated with lower acute care utilization and could provide benefits to patients, particularly those using fentanyl who might need these higher doses.

## Teaser

This cross-sectional study of US adults diagnosed with opioid use disorder examines the association of buprenorphine treatment at doses higher than FDA recommendations with subsequent acute health care utilization.

## Introduction

Opioid use disorder (OUD) affects an estimated 7 million Americans.^1^ Fatal and nonfatal opioid overdoses remain at record highs,^2^ with even greater numbers of nonfatal overdoses,^3^ and increasingly involve fentanyl, a potent synthetic opioid.^2^

Buprenorphine is one of the most effective treatments for OUD, associated with improved outcomes and decreased fatal overdoses.^4^ But as fentanyl use increases, concerns have arisen that commonly used doses of buprenorphine, such as 16 mg daily, may be insufficient for patients with OUD.^5^ Higher buprenorphine daily doses may be beneficial for individuals using fentanyl, which is a stronger μ-opioid receptor agonist than other opioids.^6^ While the FDA’s recommended target dose is 16 mg per day,^7^ observational studies have found daily buprenorphine doses above 24 mg (hereafter higher-dose) to be associated with longer treatment episodes and lower relapse rates.^8,9,10,11,12^

Despite this emerging evidence, few studies have considered how higher-dose buprenorphine affects health service utilization. To fill this gap, we examined whether higher buprenorphine doses are associated with a reduction in the likelihood of emergency department (ED) or inpatient care utilization. We focus on a behavioral health–related ED or inpatient visit as it is more likely to be associated with the current severity of a patient’s OUD than ED or inpatient use for physical health conditions. Given the association between higher doses and improved OUD treatment retention, we hypothesize that stable buprenorphine daily doses above 16 mg may be associated with a decrease in behavioral health-related ED or inpatient among patients with OUD. To test this hypothesis, we used national claims data to examine the association between an individual’s buprenorphine dosage level relative to the target dose of 16 mg per day and subsequent risk for ED or inpatient use.

## Methods

This cross-sectional study was approved by the University of Southern California institutional review board. The study follows the Strengthening the Reporting of Observational Studies in Epidemiology (STROBE) reporting guidelines.

### Data and Measures

We examined commercial claims data between 2016 and 2021 from Optum’s de-identified Clinformatics Data Mart (CDM) to examine the association between buprenorphine daily dose and ED or inpatient use. The CDM is a database of commercial claims, including Medicare Advantage enrollees, from a large insurer with coverage across 50 states. It does not include individuals with Medicaid and fee-for-service Medicare coverage. It represents about 15 million beneficiaries per year and includes information on patient demographics including race and ethnicity, and claims-level health care utilization including both health service and prescription drug utilization. The CDM includes denied claims, but excludes claims paid entirely out of pocket (eg, claims neither paid for nor denied by an insurer).

We categorized adults diagnosed with OUD who newly initiated buprenorphine treatment into buprenorphine daily dosage tiers (more than 24 mg, more than 16 mg to 24 mg, more than 8 mg to 16 mg, 1 mg to 8 mg) based on their highest, sustained buprenorphine dose; individuals had to be at a dosage level for at least 14 days to qualify for that tier. Individuals who did not receive buprenorphine for 14 or more days in any tier (927 patients) were not placed in a dosage tier (eTable 1 in Supplement 1). Buprenorphine for treatment of OUD was defined by formulation, strength, and National Drug Code identifier (eTable 3 in Supplement 1). We calculated the number of days from the first date in an individual’s highest buprenorphine dose tier to any behavioral health–related ED or inpatient visit and mean days at the highest dose tier. We excluded individuals who were not enrolled in their insurance plan for at least 90 days before their first observed dispensed buprenorphine prescription to ensure all days’ supply of prior dispensed buprenorphine had been exhausted. Information in the CDM were used to identify patient age, sex, and race and ethnicity (categorized as non-Hispanic White, non-Hispanic Black, Hispanic, and other or unknown).

To control for other factors potentially associated with ED or inpatient use, we used service claims to identify individuals with diagnoses of OUD, non-OUD substance use disorder (SUD), alcohol use disorder, mental health disorders, or physical health comorbidities in the 90-day period before initiating buprenorphine treatment (eTable 2 in Supplement 1). We also identified individuals who in the 90 days before treatment received ED or inpatient services, stratified by a primary diagnosis for a mental health disorder, a SUD, or a physical health issue; as well as individuals receiving outpatient mental health or SUD treatment or residential SUD treatment. We also controlled for relevant patterns of buprenorphine use including buprenorphine discontinuation and time from buprenorphine initiation to beginning the highest stable dose. Discontinuation was defined as 90 or more days since the expiration of buprenorphine day’s supply from the last observed dispensed buprenorphine prescription; time to dose was measured in days from buprenorphine initiation to the highest stable buprenorphine dose.

### Statistical Analysis

We conducted bivariate analyses to examine characteristics of individuals in each buprenorphine dosage tier. We then conducted survival analysis, using an accelerated failure time model with a Weibull distribution to estimate the association between buprenorphine dosage tier and time to ED or inpatient use, controlling for patient age, sex, race and ethnicity, comorbid conditions, buprenorphine utilization patterns, service utilization before buprenorphine treatment, and treatment year. We report adjusted time ratios (TR) from these models both overall and for 6-month windows to account for variations in the association over time and censoring. All findings are reported relative to the group receiving a daily dose between more than 8 mg to 16 mg, which includes the FDA target dose for maintenance treatment.^7^ Individuals were censored when they exited insurance coverage for any cause including loss of insurance and death, and at the end of our observation period. We conducted sensitivity analyses using all-cause ED or inpatient use and OUD-related ED or inpatient use as outcomes, as well as restricting the population to individuals without pre-buprenorphine ED or inpatient use. We also examined all patients dispensed buprenorphine, regardless of OUD diagnosis. Sensitivity analysis findings are comparable with the main analysis (eTable 4 in Supplement 1). Data was extracted using SAS version 9.4 (SAS Institute Inc) and analyzed using Stata version 16.0 (StataCorp LLC), results were deemed significant at *P* < .05.

## Results

We identified 35 451 unique patients with an OUD diagnosis who began buprenorphine treatment (mean [SD] age, 46.2 [15.1] years; 20 983 male [59.2%]; 3326 Black [9.4%], 2411 Hispanic [6.8%], 26 712 White [75.3%]). Patients were most likely to initiate buprenorphine in 2016 (7843 individuals [22.1%]), and 24 426 patients (68.9%) attained their maximum dose category at buprenorphine initiation. Almost half had a recent mental health diagnosis before initiating buprenorphine (17 151 patients [48.4%]); 28 643 patients (80.8%) had a recent substance use disorder diagnosis; 9904 patients (27.9%) had a recent behavioral health–related ED or inpatient visit; and 7025 patients (19.8%) had a recent physical health–related ED or inpatient visit.

Individuals in the 1 mg to 8 mg tier (9669 patients [27.3%]) received their buprenorphine dose a mean (SD) 249.5 (353.3) days; individuals in the more than 8 mg to 16 mg tier (14 802 patients [42.9%]) received their dose a mean (SD) 282.4 (379.8) days; individuals in the tier receiving more than 16 mg to 24 mg (10 329 patients [29%]) received their dose a mean (SD) 330.4 (420.9) days; and individuals in the tier receiving more than 24 mg (651 patients [1.8%]) received their dose a mean (SD) 234.0 (363.2) days. Compared with individuals in lower dosage tiers, individuals in the above 24 mg tier were more likely to be non-Hispanic White (511 of 651 patients [78.5%] vs 7117 of 9669 patients [73.6%] in the 1 mg to 8 mg tier), to have had outpatient behavioral health care in the 90 days before starting buprenorphine (468 of 651 patients [71.9%] vs 6749 of 9669 patients [69.8%] in the 1 mg to 8 mg tier), and to have started treatment in 2016 or 2017 (2016: 178 of 651 patients [27.3%] vs 1754 of 9669 patients [18.1%] in the 1 mg to 8 mg tier); they were less likely to have a diagnosis for a nonopioid SUD before beginning treatment (117 of 651 patients [18.0%] vs 2167 of 9669 patients [22.4%] in the 1 mg to 8 mg tier) (Table 1). Individuals in the above 24 mg tier took longer to achieve their highest, stable dose and were somewhat less likely to discontinue buprenorphine relative to lower dose tiers.

**Table 1.** Buprenorphine Dosage Tier Individual and Service Use Characteristics

Characteristic	Participants, No. (%)				*P* value
	>24 mg (n = 651)	>16-24 mg (n = 10 329)	>8-16 mg (n = 14 802)	1-8 mg (n = 9669)	
Age, mean (SD), y	45.5 (13.3)	47.3 (14.2)	44.4 (14.6)	48.1 (16.5)	<.001
Sex					
Female	270 (41.5)	4184 (40.5)	5606 (37.9)	4401 (45.5)	<.001
Male	381 (58.5)	6143 (59.5)	9194 (62.1)	5265 (54.5)	
Race and ethnicity					
Non-Hispanic Black	54 (8.3)	1022 (9.9)	1348 (9.1)	902 (9.3)	<.001
Hispanic	34 (5.2)	694 (6.7)	915 (6.2)	768 (7.9)	
Non-Hispanic White	511 (78.5)	7842 (75.9)	11 242 (75.9)	7117 (73.6)	
Other or unknown^a^	52 (8.0)	771 (7.5)	1297 (8.8)	882 (9.1)	
Year					
2016	178 (27.3)	2608 (25.2)	3303 (22.3)	1754 (18.1)	<.001
2017	121 (18.6)	1493 (14.5)	2143 (14.5)	1128 (11.7)	
2018	89 (13.7)	1563 (15.1)	2186 (14.8)	1343 (13.9)	
2019	89 (13.7)	1761 (17.0)	2545 (17.2)	2030 (21.0)	
2020	112 (17.2)	1477 (14.3)	2291 (15.5)	1670 (17.3)	
2021	62 (9.5)	1427 (13.8)	2334 (15.8)	1744 (18.0)	
Comorbid diagnoses					
Mental health	317 (48.7)	4976 (48.2)	6934 (46.8)	4924 (50.9)	<.001
Any substance use	515 (79.1)	8144 (78.8)	12 368 (83.6)	7616 (78.8)	<.001
Any opioid use disorder	498 (76.5)	7833 (75.8)	11 965 (80.8)	7317 (75.7)	<.001
Any nonopioid SUD	117 (18.0)	1913 (18.5)	3383 (22.9)	2167 (22.4)	<.001
Any alcohol use disorder	55 (8.4)	844 (8.2)	1500 (10.1)	1055 (10.9)	<.001
Preperiod service utilization					
ED or inpatient behavioral health use	163 (25.0)	2604 (25.2)	4257 (28.8)	2880 (29.8)	<.001
ED or inpatient physical health use	121 (18.6)	2069 (20.0)	2786 (18.8)	2049 (21.2)	<.001
Outpatient behavioral health use	468 (71.9)	7096 (68.7)	10 391 (70.2)	6749 (69.8)	.043
Residential SUD service use	31 (4.8)	406 (3.9)	935 (6.3)	609 (6.3)	<.001
Time between first fill and dose category, d					
0	123 (18.9)	4555 (44.1)	10 414 (70.4)	9334 (96.5)	<.001
1-30	70 (10.8)	1838 (17.8)	2064 (13.9)	247 (2.6)	
31-90	104 (16.0)	1249 (12.1)	844 (5.7)	37 (0.4)	
91-365	178 (27.3)	1568 (15.2)	914 (6.2)	39 (0.4)	
>365	176 (27.0)	1119 (10.8)	566 (3.8)	12 (0.1)	
Discontinuation of buprenorphine	55 (8.4)	1536 (14.9)	2770 (18.7)	2781 (28.8)	<.001
Outcome measures					
Any ED or inpatient service	113 (17.4)	2197 (21.3)	2929 (19.8)	1799 (18.6)	<.001
With behavioral health diagnosis^b^	69 (10.6)	1420 (13.7)	1877 (12.7)	1065 (11.0)	<.001
With OUD diagnosis	47 (7.2)	922 (8.9)	1308 (8.8)	670 (6.9)	<.001

Abbreviations: ED, emergency department; OUD, opioid use disorder; SUD, substance use disorder.

^a^ Other or unknown indicates a reported race and ethnicity of non-Hispanic Asian or missing or unknown.

^b^ Categories are not mutually exclusive. Detailed descriptions of measures are available in eTable 2 in Supplement 1.

In unadjusted data, OUD diagnosed individuals in both the above 24 mg buprenorphine tier and the more than 16 mg to 24 mg tier did not receive ED or inpatient services as quickly after reaching their highest buprenorphine dose as did individuals in the more than 8 mg to 16 mg tier (69.5 [95% CI, 49.2-89.8] days slower and 57.6 [95% CI, 50.8-64.3] days slower, respectively, on average) (Figure). That result was true for the above 16 mg to 24 mg group for most of the observation window as well. While the difference in use of ED or inpatient services across different dose tiers was modest in the first 6 months after buprenorphine initiation, the differences between the above 24 mg tier and other tiers become more pronounced subsequently. This is consistent with the regression-adjusted time ratios for an ED or inpatient visit, which estimate the time to the behavioral health–related ED or inpatient visit in days, conditional upon dosage categories and other controls (Table 2). These results show that patients in the above 24 mg tier and the tier receiving more than 16 mg to 24 mg tier experience longer times to an ED or inpatient service use than patients in the more than 8 mg to 16 mg tier (TR, 1.37; 95% CI, 1.81-1.04; TR, 1.11; 95% CI, 1.20-1.02, respectively). We estimate similar regression-adjusted accelerated failure time ratios but restrict the observation window to 6 months, 1 year, and so on to ascertain if any bias was found in results due to the length of time in which the patient was observed postinitiation of highest stable buprenorphine dose (Table 3). We found a similar trend of longer time to a behavioral health–related ED or inpatient visit for the above 16 mg to 24 mg group for all 6-month periods of observation (180 days: TR, 1.14; 95% CI, 1.00-1.30; 1095 days: TR, 1.12; 95% CI, 1.02-1.22). We found an even larger effect on the time to ED or inpatient visit among the more than 24 mg tier, particularly as the observation window grew, but these differences were not statistically significant until 1 year after buprenorphine initiation (TR, 1.48; 95% CI, 1.01-2.18). The 1 mg to 8 mg tier had a similar time to an ED or inpatient visit as the tier receiving 8 mg to 16 mg tier.

**Figure.**
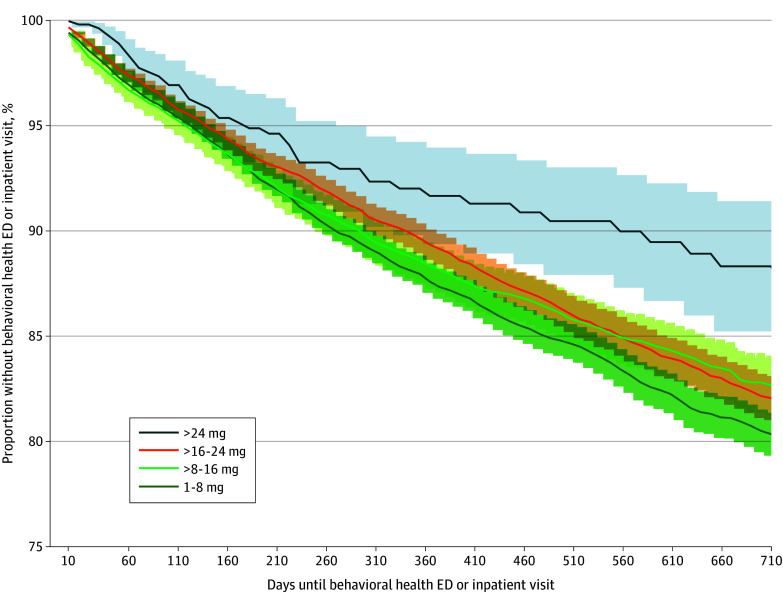
Days Until ED or Inpatient Services for Behavioral Health Diagnosis in 2 Years Following First Fill of a Patient’s Highest Stable Dose of Buprenorphine ED indicates emergency department; shaded areas represent 95% CIs. Time to ED or inpatient claim is measured in days from the first fill of the patient’s highest stable dose of buprenorphine. All patients required 90 days of enrollment prior to filling first buprenorphine prescription (at any dose). Data presented is truncated at 720 days for presentation purposes, but the observation window lasts up to 2191 days.

**Table 2.** Regression-Adjusted Accelerated Failure Time Ratio for Time to Behavioral Health ED or Inpatient Claim by Highest Buprenorphine Dose^a^

Outcome	TR (95% CI) (N = 35 451)	*P* value
Dose		
>8-16 mg	1 [Reference]	[Reference]
>24 mg	1.37 (1.04-1.81)	.02
>16-24 mg	1.11 (1.02-1.20)	.01
1-8 mg	1.03 (0.95-1.13)	.46
Race or ethnicity		
Non-Hispanic White	1 [Reference]	[Reference]
Non-Hispanic Black	0.96 (0.85-1.09)	.55
Hispanic	1.10 (0.96-1.27)	.34
Other or unknown^b^	0.82 (0.72-0.93)	.002
Age, per 1 y	1.01 (1.00-1.01)	<.001
Sex		
Female	1 [Reference]	[Reference]
Male	1.11 (1.04-1.19)	.003
Year		
2019	1 [Reference]	[Reference]
2016	0.84 (0.75-0.93)	.001
2017	1.15 (1.01-1.31)	.03
2018	1.09 (0.96-1.24)	.18
2020	0.88 (0.76-1.01)	.06
2021	0.88 (0.79-0.93)	.15
Buprenorphine use patterns		
Days to dose	1.00 (1.00-1.00)	<.001
Discontinuation	2.95 (2.69-3.25)	<.001
Diagnosis		
No relevant diagnosis in 90 d prior	1 [Reference]	[Reference]
Alcohol use disorder	0.98 (0.81-1.18)	.85
Opioid use disorder	1.10 (1.01-1.20)	.03
Nonopioid SUD	0.84 (0.76-0.94)	.002
Mental health	0.86 (0.76-0.93)	<.001

Abbreviations: ED, emergency department; SUD, substance use disorder; TR, time ratio.

^a^ Time to behavioral health ED or inpatient claim is measured in days from the first fill of the patient’s highest stable dose of buprenorphine. All patients required 90 days of enrollment prior to being dispensed first buprenorphine prescription (at any dose). Regressions also control for time from behavioral health ED or inpatient visit to start of buprenorphine, time from residential substance use treatment to start of buprenorphine, outpatient behavioral health use in 90 days prior to filling buprenorphine, time from initiation of buprenorphine to achieving highest dose, discontinuation of buprenorphine, and any physical health ED or inpatient visit in 90 days prior to filling first buprenorphine prescription. Complete results available in eTable 4 in Supplement 1.

^b^ Other or unknown indicates a reported race or ethnicity of non-Hispanic Asian or missing or unknown race or ethnicity.

**Table 3.** Time-Varying Accelerated Failure Time Ratios (TRs) for Time to Behavioral Health–Related ED or Inpatient Claim by Patient’s Highest Buprenorphine Dose and Observation Window^a^

Dose category	Observation window, TR (95% CI)					
	180 d	365 d	545 d	730 d	1095 d	Full window
>8-16 mg	1 [Reference]	1 [Reference]	1 [Reference]	1 [Reference]	1 [Reference]	1 [Reference]
>24 mg	1.36 (0.86-2.15)	1.48 (1.01-2.18)	1.62 (1.11-2.35)	1.64 (1.14-2.34)	1.53 (1.11-2.11)	1.37 (1.04-1.81)
*P* value	.19	.05	.01	.01	.01	.02
>16-24 mg	1.14 (1.00-1.30)	1.19 (1.06-1.32)	1.15 (1.04-1.27)	1.15 (1.04-1.26)	1.12 (1.02-1.22)	1.11 (1.02-1.20)
*P* value	.04	.00	.01	.01	.02	.01
1-8 mg	0.86 (0.76-0.98)	0.93 (0.84-1.04)	0.97 (0.87-1.08)	1.00 (0.90-1.10)	1.01 (0.92-1.11)	1.03 (0.95-1.13)
*P* value	.02	.22	.57	.95	.83	.46

Abbreviation: ED, emergency department.

^a^ Time to behavioral health ED or inpatient claim is measured in days from the first fill of the patient’s highest stable dose of buprenorphine. All patients required 90 days of enrollment prior to being dispensed first buprenorphine prescription (at any dose). Regressions also control for age, sex, race and ethnicity, year of index dose, pre-buprenorphine comorbidities, time from behavioral health ED or inpatient visit to start of buprenorphine, time from residential substance use treatment to start of buprenorphine, outpatient behavioral health use in 90 days prior to filling buprenorphine, time from initiation of buprenorphine to achieving highest dose, discontinuation of buprenorphine, and any physical health ED or inpatient visit in 90 days prior to filling first buprenorphine prescription. Complete results available in eTable 4 in Supplement 1.

We also found longer times to ED or inpatient services among male patients (TR, 1.11; 95% CI, 1.04-1.09) and individuals with an OUD diagnosis prior to initiating buprenorphine (TR, 1.10; 95% CI, 1.01-1.20); age was negatively associated with time to ED or inpatient services (TR, 1.01; 95% CI, 1.00-1.01) (Table 2). In contrast, individuals with a prior diagnosis of a nonopioid SUD (TR, 0.84; 95% CI, 0.76-0.94) or a comorbid mental health disorder (TR, 0.86; 95% CI, 0.76-0.93) had faster times to ED or inpatient services. Individuals who ended their buprenorphine episodes also had longer times to ED or inpatient services (TR, 2.95; 95% CI, 2.96-3.25). Findings were comparable when we examined time to an OUD diagnosis–related ED or inpatient service, and when we restricted analyses to individuals with no observed ED or inpatient visit in the 90 days before treatment (eTable 5 in Supplement 1).

## Discussion

Despite emerging evidence that buprenorphine dosages exceeding 16 mg and 24 mg may be beneficial, there has been little examination of how such dosages may affect urgent health care service utilization. Our exploratory analysis of commercially insured individuals receiving buprenorphine, including Medicare Advantage beneficiaries, found that individuals who receive buprenorphine treatment with stable doses above 24 mg, as well as individuals receiving stable doses of more than 16 mg to 24 mg, may have lower risk for subsequent ED or inpatient utilization than individuals receiving stable doses of more than 8 mg to 16 mg. Daily doses of more 8 mg to 16 mg are more common than higher doses,^6,13,14^ likely influenced to some extent by both state laws, insurance policies and federal language regarding 16 mg as a target dose.^7,15,16^ While there is increasing attention to the potential benefits of increasing access to buprenorphine through low-barrier and targeted programs to historically underserved populations,^17,18,19^ such as individuals from racial and ethnic minority populations, incarcerated individuals, and pregnant people,^20,21,22^ our findings contribute to the growing research regarding the potential benefits of higher buprenorphine doses, and suggest revisiting policies and guidelines that may serve as barriers to higher doses should also be part of these conversations.

While it is often referred to as the opioid crisis, there has been increasing recognition that it is common for individuals with OUD to also be misusing other substances,^2,23,24^ and that it would be more accurate to refer to the current situation as a polysubstance crisis.^25^ Similarly, high rates of mental health comorbidity in individuals with OUD has long been documented.^26^ These populations also appear to have higher rates of ED or inpatient utilization, suggesting the complexities of addressing these comorbid disorders while treating OUD, and the need for the delivery of effective interventions to treat such comorbid disorders concurrently.

### Limitations

Our findings must be considered within the context of study limitations. We do not know if our findings would generalize to Medicaid enrollees or other populations. We use pharmacy claims to identify when individuals receive the highest stable buprenorphine dose, and are unable to observe dispensed buprenorphine not paid for by insurance. We also do not know to what extent the emergence and growth in use of long-acting buprenorphine formulations in recent years might influence the highest stable dose received by patients on buprenorphine. We have no clinical information, including OUD severity, or clinical history before enrollment in Optum. In addition, we lack clinical information on severity of withdrawal symptoms, detailed substance use histories, and other clinical markers. Such clinical factors likely influence buprenorphine dose, subsequent service utilization, or the ending of buprenorphine treatment, and we do not know to what extent such unmeasured confounding may influence the reliability of our results. Electronic health records containing both clinical and pharmacy information are likely better suited to examine these complex relationships. As we cannot control for all available confounding, we treat our findings as associational rather than causal.

Our key comparison is with a patient’s maximum stable dose of buprenorphine, and although we directly control for the days it takes for a patient to achieve that does, it may not fully control for immortal time bias, making comparisons of patients on the highest dose with those on the lowest dose affected by selection bias. We believe that directly controlling for the time it takes for a patient to achieve maximum stable dose mitigates the risk of immortal time bias, while acknowledging it remains a concern that should be considered and addressed in future research.

## Conclusions

The results of this cross-sectional study contribute to the sparse empirical research regarding the potential benefits of the use of higher doses of buprenorphine in individuals with OUD. To the extent buprenorphine prescribing practices may be evolving in response to growth in use of fentanyl, it is important to consider the potential effects on health care use and clinical outcomes, as well as to ensure equitable access to individuals who could potentially benefit from higher doses. Specifically, it will be important to understand to what extent barriers, such as state laws^15^ and Medicaid policies^27^ that limit doses of buprenorphine for OUD treatment to 16 mg or 24 mg, might curtail access for some vulnerable individuals.

## Notes

Supplement 1. **eTable 1.** Sample Selection Details **eTable 2.** Definitions Used in the Construction of Sample, Covariates, and Outcomes of Interest **eTable 3.** List of NDCs and Generic Drug Names Identified as Buprenorphine **eTable 4.** Full Regression Output for In-Text Table 2 **eTable 5.** Alternative Specifications **eTable 6.** Model Selection **eFigure.** Days Until All-Cause ED or Inpatient Services From First Fill of the Patient’s Highest Stable Dose of Buprenorphine

Supplement 2.
Data Sharing Statement
