# Supplementary material for: Association of Daily Doses of Buprenorphine With Urgent Health Care Utilization
Source: JAMA Netw Open. 2024 Sep 25;7(9):e2435478. doi: 10.1001/jamanetworkopen.2024.35478 (PMC11425142; doi:10.1001/jamanetworkopen.2024.35478)
Supplement: Supplement 1. — eTable 1. Sample Selection Details eTable 2. Definitions Used in the Construction of Sample, Covariates, and Outcomes of Interest eTable 3. List of NDCs and Generic Drug Names Identified as Buprenorphine eTable 4. Full Regression Output for In-Text Table 2 eTable 5. Alternative Specifications eTable 6. Model Selection eFigure. Days Until All-Cause ED or Inpatient Services From First Fill of the Patient’s Highest Stable Dose of Buprenorphine [file jamanetwopen-e2435478-s001.pdf]

Supplemental Online Content

Axeen S, Pacula RL, Merlin JS, Gordon AJ, Stein BD. Association of daily doses of buprenorphine with urgent health care utilization. *JAMA Netw Open.* 2024;7(9):e2435478. doi:10.1001/jamanetworkopen.2024.35478

eTable 1. Sample Selection Details

eTable 2. Definitions Used in the Construction of Sample, Covariates, and Outcomes of Interest

eTable 3. List of NDCs and Generic Drug Names Identified as Buprenorphine

eTable 4. Full Regression Output for In-Text Table 2

eTable 5. Alternative Specifications

eTable 6. Model Selection

eFigure. Days Until All-Cause ED or Inpatient Services From First Fill of the Patient's Highest Stable Dose of Buprenorphine

This supplemental material has been provided by the authors to give readers additional information about their work.

© 2024 Axeen S et al. *JAMA Network Open.*

eTable 1. Sample Selection Details

Selection Criteria	Unique Patients	Share of Prior Category
	N	%
All Buprenorphine Users, 2016 - 2021	102,503	
Enrolled 90 Days Before First Buprenorphine Fill	55,557	54.2
At Least 14 Days of Buprenorphine	54,630	98.3
Diagnosis of Opioid Use Disorder+	35,451	64.9
Final Sample	35,451	

Notes: + Presence of ICD-10 Codes F11* during the patient's observation window (90 days prior to treatment initiation until disenrollment).

© 2024 Axeen S et al. *JAMA Network Open.*

eTable 2. Definitions Used in the Construction of Sample, Covariates, and Outcomes of Interest

Site of Care	Definition
Emergency Department	Procedure codes: 99281 – 99285<br>OR<br>Place of service: "Emergency Room"<br>OR<br>Revenue codes: 0450, 0451 ,0452, 0456, 0459, 0981
Inpatient	Place of Service: "Inpatient Hospital"<br>OR<br>Presence of a confinement code indicating a hospital-based stay
Outpatient	Place of service: "Office/Clinic"
Residential Substance Use	Place of service: "Residential Substance Abuse Treatment Facility"
Diagnoses of Interest in 90 Days Prior to Treatment Initiation	
Any Behavioral Health	Any Mental Health OR Any Substance Use Disorder as defined below
Any Mental Health	ICD 10 Codes: F2*, F3*, F4*, F50*, F53*, F60*, F63*, F90*, F91* in 90 days prior to treatment initiation
Any Substance Use Disorder	Any opioid-related substance use disorder, any alcohol-related substance use disorder, any drug-related substance use disorder as defined below in 90 days prior to treatment initiation
Opioid-Related Substance Use Disorder	ICD 10 Codes: F11* in 90 days prior to treatment initiation
Alcohol-Related Substance Use Disorder	ICD 10 Codes: F10* in 90 days prior to treatment initiation
Any Non-Opioid Drug-Related Substance Use Disorder	ICD 10 Codes: F12*, F13*, F14*, F15*, F16*, F18*, F19* in 90 days prior to treatment initiation
Physical Health Encounters	Elixhauser codes for: Congestive heart failure, cardiac arrhythmias, valvular disease, pulmonary circulation disorders, peripheral vascular disorders, hypertension (complicated and uncomplicated), paralysis, other neurological disorders, COPD, Diabetes, hypothyroidism, renal failure, liver disease, peptic ulcer disease, aids/hiv, lymphoma, metastatic cancer, solid tumor without metastasis, rheumatoid arthritis, coagulopathy, obesity, weight loss, fluid and electrolyte disorders, blood loss anemia, or deficiency anemia in 90 days prior to treatment initiation

© 2024 Axeen S et al. *JAMA Network Open.*

eTable 3. List of NDCs and Generic Drug Names Identified as Buprenorphine

Generic Name	Strength	NDCs
Buprenorphine HCL	2 MG	00054017613, 00093537856, 00228315603, 00378092393, 42858050103, 50383092493, 62756045983
	8 MG	00054017713, 00093537956, 00228315303, 00378092493, 42858050203, 43063075306, 50383093093, 60687049221, 62756046083
Buprenorphine HCL/ Naloxone HCL	0.7-0.18MG	54123090730
	1.4-0.36MG	54123091430
	11.4-2.9MG	54123011430
	12 MG-3 MG	00378876816, 00378876893, 00781724964, 12496121201, 12496121203, 43598058101, 43598058130, 47781035803, 47781035811, 52427071203
	2 MG-0.5MG	00054018813, 00093572056, 00228315403, 00228315473, 00378876516, 00378876593, 00406192303, 00406800503, 00781721606, 00781721664, 00904700906, 12496120201, 12496120203, 16729054910, 42291017430, 42858060103, 43598057901, 43598057930, 47781035503, 47781035511, 50383029493, 52427069203, 52427069211, 60429058630, 60429058633, 62175045232, 62756096983, 65162041603
	2.1-0.3 MG	59385001230
	2.9-0.71MG	54123092930
	4.2-0.7 MG	59385001401, 59385001430
	4MG-1MG	00378876616, 00378876693, 00781722764, 12496120401,12496120403, 43598058001, 43598058030, 47781035603, 47781035611
	5.7-1.4 MG	54123095730
	6.3MG-1MG	59385001601, 59385001630
	8 MG-2 MG	00054018913, 00093572156, 00228315503, 00228315567, 00228315573, 00378876716, 00378876793, 00406192403, 00406802003, 00781723806, 00781723864, 12496120801, 12496120803, 16729055010, 42291017530, 42858060203, 43598058201, 43598058230, 47781035703, 47781035711, 50268014515, 50383028793, 52427069803, 52427069811, 60429058730, 60429058733, 62175045832, 62756097083, 65162041503
	8.6-2.1 MG	54123098630

© 2024 Axeen S et al. *JAMA Network Open.*

eTable 4. Full Regression Output for In-Text Table 2

	Time Ratio	p-value	95% CI
Dose Category			
>24 MG	1.37	0.01	1.04-1.81
>16-24 MG	1.11	0.02	1.02-1.20
>8-16 MG			
1-8 MG	1.03	0.83	0.95-1.13
Age	1.01	<0.01	(1.00 - 1.01
Race/Ethnicity			
NH-Black	0.96	0.55	0.85-1.09
Hispanic	1.10	0.34	0.96-1.27
Other/Unknown	0.82	<0.01	0.72-0.93
Sex			
Male	1.11	<0.01	1.04-1.19
Year			
2016	0.84	<0.01	0.75-0.93
2017	1.15	0.03	1.01-1.31
2018	1.09	0.18	0.96-1.24
2019			
2020	0.88	0.06	0.76-1.01
2021	0.88	0.15	0.79-0.93
Pre-Period Comorbidity			
Alcohol SUD	0.98	0.85	0.81-1.18
Opioid SUD	1.10	0.03	1.01-1.20
Non-Opioid SUD	0.84	<0.01	0.76-0.94
Mental Health Dx	0.86	<0.01	0.76-0.93
Days from pre-period BH ED/IP to Bupe			
1-7 days	0.56	0.31	0.66-3.01
8-14 days	0.94	0.94	0.30-1.96
15-30 days	1.06	0.94	0.73-10.91
31-90 days	0.92	0.91	0.92-5.57
No visit	0.05	<0.01	0.67-1.64
Days from pre-period Residential SUD Stay to Bupe			
1-7 days	1.42	0.27	0.66-3.02
8-14 days	0.76	0.56	0.30-1.96
15-30 days	2.82	0.14	0.73-10.91

© 2024 Axeen S et al. *JAMA Network Open.*

31-90 days	2.27	0.08	0.92-5.57
No visit	1.05	0.84	0.67-1.64
Pre-Period Utilization			
Any OP BH Visit	1.06	0.23	0.97-1.16
Any PH ED/IP Visit	69.80	<0.01	28.06-173.59
Days to Dose	1.00	<0.01	1.00-1.00
Discontinue	2.95	<0.01	2.69-3.25
N	35,451		
Likelihood Ratio	2418.11		
p-value	<0.01		

Notes: Time to behavioral health ED or Inpatient Claim is measured in days from the first fill of the patient's highest stable dose of buprenorphine. All patients required 90 days of enrollment prior to being dispensed first buprenorphine prescription (at any dose).

*Other/Unknown indicates a reported race/ethnicity of Non-Hispanic Asian or a missing or unknown race/ethnicity.

© 2024 Axeen S et al. *JAMA Network Open.*

eTable 5. Alternative Specifications

Type of ED/IP Visit	BH	BH	All Cause	OUD ED/IP
Sample	All Users<br>N=50,380	No ED/IP Visit in Pre-Period<br>N=25,547	Baseline<br>N=35,451	Baseline<br>N=35,451
	Time Ratio<br>(95% CI)	Time Ratio<br>(95% CI)	Time Ratio<br>(95% CI)	Time Ratio<br>(95% CI)
Dose				
>24 MG	1.152<br>(0.932 - 1.425)	1.369**<br>(1.042 - 1.799)	1.243*<br>(0.989 - 1.561)	1.395**<br>(1.020 - 1.908)
>16-24 MG	1.054<br>(0.986 - 1.127)	1.116***<br>(1.031 - 1.208)	1.067*<br>(0.997 - 1.142)	1.153***<br>(1.052 - 1.263)
>8-16 MG	Ref	Ref	Ref	Ref
1-8 MG	1.147***<br>(1.067 - 1.234)	1.025<br>(0.940 - 1.117)	1.006<br>(0.936 - 1.080)	1.094*<br>(0.990 - 1.210)
Age	1.004***<br>(1.002 - 1.006)	1.010***<br>(1.007 - 1.012)	1.000<br>(0.998 - 1.003)	1.016***<br>(1.013 - 1.019)
Race/Ethnicity				
White	Ref	Ref	Ref	Ref
NH-Black	1.005<br>(0.906 - 1.114)	0.974<br>(0.864 - 1.099)	0.963<br>(0.871 - 1.066)	1.064<br>(0.922 - 1.228)
Hispanic	1.120*<br>(0.996 - 1.260)	1.127*<br>(0.979 - 1.296)	1.065<br>(0.948 - 1.196)	1.057<br>(0.902 - 1.240)
Other/Unknown	0.838***<br>(0.749 - 0.937)	0.819***<br>(0.719 - 0.933)	0.870**<br>(0.779 - 0.971)	0.862*<br>(0.743 - 1.001)
Sex				
Male	1.152***<br>(1.086 - 1.221)	1.101***<br>(1.027 - 1.180)	1.140***<br>(1.075 - 1.209)	0.971<br>(0.895 - 1.054)
Female	Ref	Ref	Ref	Ref
Year				
2016	0.749***<br>(0.683 - 0.822)	0.796***<br>(0.716 - 0.885)	0.709***<br>(0.647 - 0.777)	0.750***<br>(0.664 - 0.847)
2017	1.088<br>(0.971 - 1.219)	1.131*<br>(0.995 - 1.285)	0.963<br>(0.864 - 1.074)	1.257***<br>(1.084 - 1.457)
2018	1.094<br>(0.975 - 1.227)	1.091<br>(0.959 - 1.241)	0.949<br>(0.851 - 1.058)	1.105<br>(0.955 - 1.278)
2019	Ref	Ref	Ref	Ref
2020	0.922<br>(0.815 - 1.042)	0.886*<br>(0.770 - 1.019)	0.885**<br>(0.786 - 0.997)	0.880<br>(0.749 - 1.034)
2021	0.849**<br>(0.734 - 0.983)	0.884<br>(0.744 - 1.051)	0.886*<br>(0.769 - 1.020)	0.768***<br>(0.635 - 0.930)
Discontinuation	2.469***<br>(2.248 - 2.711)	3.006***<br>(2.732 - 3.308)	1.644***<br>(1.533 - 1.762)	2.810***<br>(2.498 - 3.160)
Days to Dose	1.001***<br>(1.001 - 1.001)	1.000**<br>(1.000 - 1.001)	1.001***<br>(1.001 - 1.002)	1.002***<br>(1.001 - 1.002)
Pre-Period Dx				
Alcohol UD	0.944<br>(0.786 - 1.134)	0.950<br>(0.788 - 1.147)	1.074<br>(0.910 - 1.266)	0.875<br>(0.714 - 1.071)
OUD	0.683***	1.103**	1.052	1.134**

© 2024 Axeen S et al. *JAMA Network Open.*

	(0.632 - 0.738)	(1.011 - 1.202)	(0.977 - 1.133)	(1.025 - 1.255)
Substance UD	0.791***<br>(0.711 - 0.881)	0.848***<br>(0.760 - 0.946)	1.029<br>(0.933 - 1.135)	0.899*<br>(0.794 - 1.017)
Mental Health	0.845***<br>(0.783 - 0.912)	0.868***<br>(0.803 - 0.938)	0.928**<br>(0.868 - 0.991)	1.068<br>(0.974 - 1.171)
Likelihood Ratio	2672.16	1032.66	3223.99	2723.08
p-value	<0.01	<0.01	<0.01	<0.01

Notes: Time to BH ED or Inpatient Claim is measured in days from the first fill of the patient's highest stable dose of buprenorphine. All patients required 90 days of enrollment prior to being dispensed first buprenorphine prescription (at any dose).

*** p<0.01, ** p<0.05, *p>0.1

© 2024 Axeen S et al. *JAMA Network Open.*

eTable 6. Model Selection

VARIABLES	1	2	3	4
>24 MG	1.373**	1.489***	1.715***	1.224
	(1.043 - 1.806)	(1.126 - 1.969)	(1.307 - 2.251)	(0.923 - 1.624)
>16-24 MG	1.108**	1.096**	1.186***	1.033
	(1.024 - 1.199)	(1.011 - 1.188)	(1.097 - 1.283)	(0.952 - 1.120)
>8-16 MG	reference	reference	reference	reference
1-8 MG	1.033	1.117**	0.988	1.161***
	(0.948 - 1.126)	(1.023 - 1.220)	(0.907 - 1.077)	(1.062 - 1.268)
Days from Buprenorphine to Dose	1.001***			1.001***
	(1.001 - 1.002)			(1.001 - 1.002)
Discontinuation of Buprenorphine	2.954***		2.874***	
	(2.685 - 3.251)		(2.613 - 3.163)	
Observations	35,451	35,451	35,451	35,451

95% CI of AFT Time Ratio in parentheses
*** p<0.01, ** p<0.05, * p<0.1

© 2024 Axeen S et al. *JAMA Network Open.*

eFigure. Days Until All-Cause ED or Inpatient Services From First Fill of the Patient's Highest Stable Dose of Buprenorphine

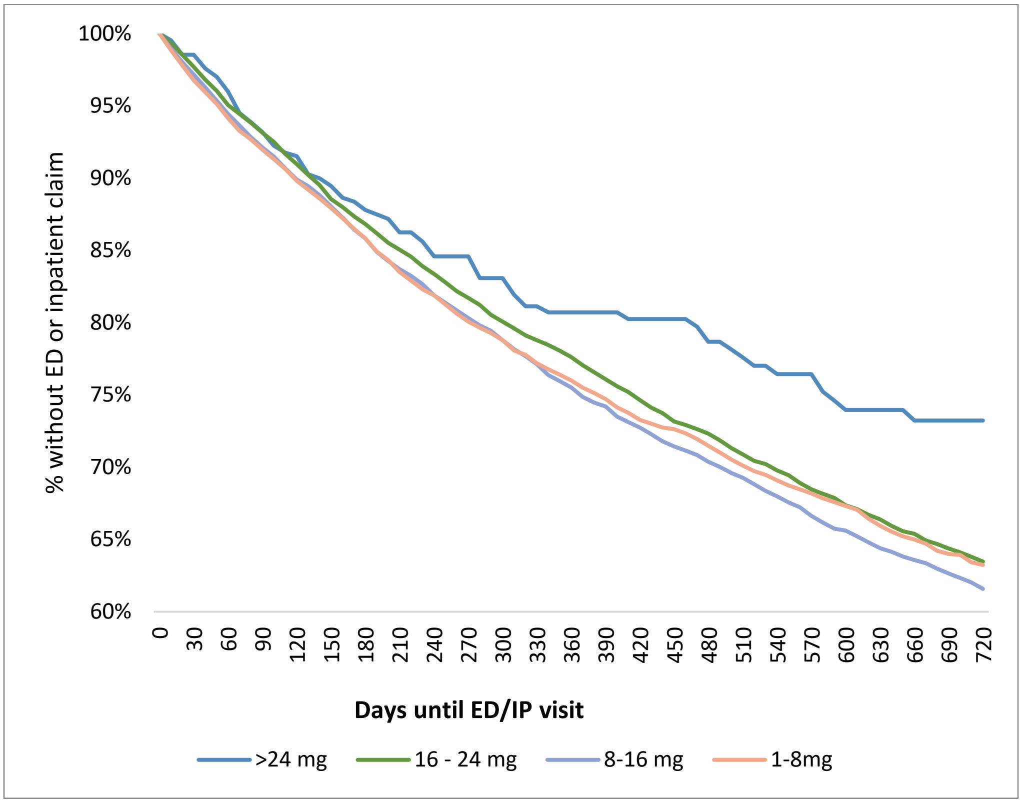

Notes: Data is truncated at 720 days for display purposes, actual observations range from [0, 2191] days. Time to ED or Inpatient Claim is measured in days from the first fill of the patient's highest stable dose of buprenorphine. All patients required 90 days of enrollment prior to filling first buprenorphine prescription (at any dose).

© 2024 Axeen S et al. *JAMA Network Open.*
