# Supplementary material for: Association of Daily Doses of Buprenorphine With Urgent Health Care Utilization
Source: JAMA Netw Open. 2024 Sep 25;7(9):e2435478. doi: 10.1001/jamanetworkopen.2024.35478 (PMC11425142; doi:10.1001/jamanetworkopen.2024.35478)
Supplement: Supplement 2. — Data Sharing Statement [file jamanetwopen-e2435478-s002.pdf]

**Data Sharing Statement**

**Data**
**Data available:** No

**Additional Information**
**Explanation for why data not available:** Data under DUA
